# Supplementary material for: A scoping review of Deaf awareness programs in Health professional education
Source: PLOS Glob Public Health. 2024 Aug 19;4(8):e0002818. doi: 10.1371/journal.pgph.0002818 (PMC11332937; doi:10.1371/journal.pgph.0002818)
Supplement: S1 Text — (DOCX) [file pgph.0002818.s002.docx]

Cinahl

Via EBSCOhost

Search date: 07/09/22

Results: 2723

| #1 | ( deaf or hard of hearing or hearing impaired or d/hh ) OR deaf aware* OR ( deaf culture or deaf communit* ) | (12,093) |
| --- | --- | --- |
| #2 | (MH "Deafness+") | (8,541) |
| #3 | S1 OR S2 | (13,711) |
| #4 | learn* OR educat* OR train* OR course* OR program* OR teach* | (1,638,361) |
| #5 | (MH "Deaf Education") | (1,261) |
| #6 | (MH "Education+") OR (MH "Education, Interdisciplinary") OR (MH "Education, Pharmacy Technicians") OR (MH "Education, Nursing, Diploma Programs") OR (MH "Education, Pharmacy") OR (MH "Education, Nursing, Associate") OR (MH "Outcomes of Education") OR (MH "Education, Non-Traditional+") OR (MH "Education, Emergency Medical Services") OR (MH "Education, Physical Education") OR (MH "Education, Podiatry") | (1,014,114) |
| #7 | "deaf awareness" | (31) |
| #8 | S4 OR S5 OR S6 OR S7 | (1,914,607) |
| #9 | S3 AND S8 | (6,290) |
| #10 | Limited to 2000-2022, English Language, peer-reviewed, research article | (2723) |
