# Supplementary material for: A scoping review of Deaf awareness programs in Health professional education
Source: PLOS Glob Public Health. 2024 Aug 19;4(8):e0002818. doi: 10.1371/journal.pgph.0002818 (PMC11332937; doi:10.1371/journal.pgph.0002818)
Supplement: Attachment — Submitted filename: Response to reviewers.docx [file pgph.0002818.s003.docx]

PGPH-D-23-02572
A Scoping Review of Deaf Awareness Programs in Health Professional Education
PLOS Global Public Healthy

Response to reviewer and editor: Thank you for your helpful comments. The changes are highlighted in yellow in the manuscript.

1. We noticed you have some minor occurrence of overlapping text with the following previous publication(s), which needs to be addressed:

- <https://doi.org/10.1111/tct.13304>

Response: Thanks. This refers to Ham et al paper in Table 1, text now paraphrased.

- <https://doi.org/10.1111/j.1365-2929.2005.02153.x>

Response: Thanks this refers to O’Neill et al paper in Table 1, text now paraphrased.

In your revision ensure you cite all your sources (including your own works), and quote or rephrase any duplicated text outside the methods section. Further consideration is dependent on these concerns being addressed.

Response: To our knowledge sources to published works are cited, including own works.

2. Please send a completed 'Competing Interests' statement, including any COIs declared by your co-authors. If you have no competing interests to declare, please state "The authors have declared that no competing interests exist".

Response: Thanks, now added.

3. "Please provide a/amend your detailed Financial Disclosure statement. This is published with the article. It must therefore be completed in full sentences and contain the exact wording you wish to be published.

a. State the initials, alongside each funding source, of each author to receive each grant.

Response: Thanks, we note most journal papers use an author contributions section, so we have now included one, and author initials in the Funding details section.

b. State what role the funders took in the study. If the funders had no role in your study, please state: “The funders had no role in study design, data collection and analysis, decision to publish, or preparation of the manuscript.”

Response: Thanks, as funders had no role, this text has now been added.

4. Please provide separate figure files in .tif or .eps format and remove the embedded figures from the manuscript file.

For more information about figure files please see our guidelines:

https://journals.plos.org/globalpublichealth/s/figures 
https://journals.plos.org/globalpublichealth/s/figures#loc-file-requirements

Response: Both figures have been removed from manuscript, and now one uploaded as a supplementary file, and the other removed. At present we were unsure about the Table, so have left this within the actual manuscript as guidance referred only to figures.

5. Some material included in your submission may be copyrighted. According to PLOS’s copyright policy, authors who use figures or other material (e.g., graphics, clipart, maps) from another author or copyright holder must demonstrate or obtain permission to publish this material under the Creative Commons Attribution 4.0 International (CC BY 4.0) License used by PLOS journals. Please closely review the details of PLOS’s copyright requirements here: PLOS Licenses and Copyright. If you need to request permissions from a copyright holder, you may use PLOS's Copyright Content Permission form.

Response: Thanks, the PRISMA image used is a free downloadable template that is in the public domain - <http://prisma-statement.org/documents/PRISMA%202009%20flow%20diagram.pdf>

Potential Copyright Issues:

Figure 2: please (a) provide a direct link to the base layer of the map (i.e., the country or region border shape) and ensure this is also included in the figure legend; and (b) provide a link to the terms of use / license information for the base layer image or shapefile. We cannot publish proprietary or copyrighted maps (e.g. Google Maps, Mapquest) and the terms of use for your map base layer must be compatible with our CC-BY 4.0 license.

Note: if you created the map in a software program like R or ArcGIS, please locate and indicate the source of the basemap shapefile onto which data has been plotted.

If your map was obtained from a copyrighted source please amend the figure so that the base map used is from an openly available source. Alternatively, please provide explicit written permission from the copyright holder granting you the right to publish the material under our CC-BY 4.0 license.

Please note that the following CC BY licenses are compatible with PLOS license: CC BY 4.0, CC BY 2.0 and CC BY 3.0, meanwhile such licenses as CC BY-ND 3.0 and others are not compatible due to additional restrictions.

If you are unsure whether you can use a map or not, please do reach out and we will be able to help you. The following websites are good examples of where you can source open access or public domain maps: 
* U.S. Geological Survey (USGS) - All maps are in the public domain. (http://www.usgs.gov) 
* PlaniGlobe - All maps are published under a Creative Commons license so please cite “PlaniGlobe, http://www.planiglobe.com, CC BY 2.0” in the image credit after the caption. (http://www.planiglobe.com/?lang=enl) 
* Natural Earth - All maps are public domain. (<http://www.naturalearthdata.com/about/terms-of-use/>)

Response: Thanks, on reflection the map we had included as Figure 2 was created using Microsoft Excel software, but it shows very little, so we have removed it.

6. We note that your Data Availability Statement is currently as follows: "Data is provided in the figures, table and additional files."
 
 Please confirm at this time whether or not your submission contains all raw data required to replicate the results of your study. Authors must share the “minimal data set” for their submission. PLOS defines the minimal data set to consist of the data required to replicate all study findings reported in the article, as well as related metadata and methods (https://journals.plos.org/plosone/s/data-availability#loc-minimal-data-set-definition).
 
 For example, authors should submit the following data:
 
 - The values behind the means, standard deviations and other measures reported;
 - The values used to build graphs;
 - The points extracted from images for analysis.
 
 Authors do not need to submit their entire data set if only a portion of the data was used in the reported study.
 
 If your submission does not contain these data, please either upload them as Supporting Information files or deposit them to a stable, public repository and provide us with the relevant URLs, DOIs, or accession numbers. For a list of recommended repositories, please see https://journals.plos.org/plosone/s/recommended-repositories.
 
 If there are ethical or legal restrictions on sharing a de-identified data set, please explain them in detail (e.g., data contain potentially sensitive information, data are owned by a third-party organization, etc.) and who has imposed them (e.g., an ethics committee). Please also provide contact information for a data access committee, ethics committee, or other institutional body to which data requests may be sent. If data are owned by a third party, please indicate how others may request data access.

Response: Thanks, data is now submitted as supporting information files, and this is reported at the foot of the paper.

7. We have noticed that you have uploaded Supporting Information files, but you have not included a list of legends. Please add a full list of legends for your Supporting Information files after the references list.

 Response: Thanks, we have now added a Supporting information files section.

Reviewers' comments:

5. Review Comments to the Author

Reviewer #1: I would recommend a major revision – given the importance of the topic area, rather than rejection. However, lots of work is required to make this manuscript publication ready.

Overall/General comments:
• This is an under-studied area, and challenges for Deaf people accessing healthcare services are important to highlight and consider approaches for change. As such, it could be an important contribution to the debates on access, inclusion and equity.
• I commend the authors for the co-participation of a Deaf and hard of hearing steering group

Response: Thank you for your supportive words. All our projects involve working closely with Deaf individuals and communities, with activities Deaf-led where possible.

• Methods: The team appear to have taken a rigorous approach to screening and reported this according to guidelines. However, it is not clear from the search strategy how health professional education was brought in, or whether this was filtered in the title/abstract review stage. List of databases accessed should be provided.

Response: Thanks. The PICO framework is now mentioned, so health professional education can be seen as the C for context. We had included the databases as part of the Search strategy but have now put this nearer the start of the Search strategy section so it is more visible. As it was a scoping review we did not narrow search terms with ‘health professional education’ but used terms related to learning, education, training, courses, programs and teaching, which are included in the search strategy.

• As currently written, the manuscript is wordy and difficult to follow. I would suggest more clear paragraphs in introduction and discussion that bring together fewer themes with stronger organization.
o For example, introduction could have the following 3 paragraph themes:
♣ What is known about provider-oriented disability awareness and access support programmes (what has worked, for whom, where)
♣ Challenges to access for Deaf and hard of hearing patients in health and how this is exacerbated by gaps in provider awareness
♣ How this leads to the gap in knowledge around Deaf awareness programmes for health professionals that made this study

Response: Thanks, this is helpful. The introduction section has been revised, and the three suggested sections have been included in order as suggested.

o Similarly the discussion needs to be organized around key themes that are supported by the paper’s findings; consider including some recommendations and bringing that out separately from the discussion.

Response: Thank you. The discussion has now been revised and recommendations added.

• Results: this section is organized around the type of health professional students receiving the intervention. Please go back and instead consider thematic analysis of the findings showing what is consistent or variable across the different types of programmes / students / settings

Response: Thank you. We have now revised and organised the discussion section around four deductive themes, and believe it to be more readable.

• I note that the funder is simply stated as ‘xxxx’ – please edit.

Response: Thanks this has now been revised.
