# Supplementary material for: A scoping review of Deaf awareness programs in Health professional education
Source: PLOS Glob Public Health. 2024 Aug 19;4(8):e0002818. doi: 10.1371/journal.pgph.0002818 (PMC11332937; doi:10.1371/journal.pgph.0002818)
Supplement: Attachment — Submitted filename: Response to reviewers 060624.docx [file pgph.0002818.s004.docx]

PGPH-D-23-02572R1
A Scoping Review of Deaf Awareness Programs in Health Professional Education
PLOS Global Public Health

Reviewer #2: This scoping review contributes towards important research about awareness in future medical professionals about diverse patient needs and is a great approach for designing an awareness program in Wales in the future. It explores existing Deaf awareness programs for health professional students in a very effective way. The authors use a well-established framework, and transparently outline their methods. The strengths of this manuscript include a well-defined research question, a broad search strategy with diverse resources, and clear methodology using PRISMA and PICO.
Areas for improvement include acknowledging and rationale for excluding non-English studies.

Response: Thank you for your helpful comments. Agree about further rationale needed for exclusion of non-English studies, now added.

Further, the gaps for further research could be more clearly presented.
Overall, this is a well-designed scoping review with valuable insights into Deaf awareness programs. Addressing the daily challenges Deaf and hard of hearing patients face in medical contexts.

Response: Thank you. Further has been added about gaps and suggestions for further research.

„Many diverse groups are disadvantaged because of assumptions around health literacy that may relate to English not being a first language, ability to read and write, which in turn impacts on a person’s ability to understand healthcare and pharmacy directions and information“ - page 6, please clarify. This is an important point about assumptions around health literacy that could be made stronger.

Response: Thank you. Text now revised.


In the end of the introduction: „address this knowledge gap“ Does this refer to the gap in research or the gap in the knowledge of the students?

Response: Thank you. Now amended.
