# Supplementary material for: A scoping review of Deaf awareness programs in Health professional education
Source: PLOS Glob Public Health. 2024 Aug 19;4(8):e0002818. doi: 10.1371/journal.pgph.0002818 (PMC11332937; doi:10.1371/journal.pgph.0002818)
Supplement: Attachment — Submitted filename: Response to reviewers.docx [file pgph.0002818.s005.docx]

Response to reviewers:

PGPH-D-23-02572R2:

Reviewer #2: Thank you for attending to all prior comments. I strongly belief this is very important research and adds value to the research corpus. As I was reviewing your paper again, I found a few paragraphs that were not consistent in the presentation of the thought process. Concluding sentences in the end of the paragraph suddenly introduced new ideas. It becomes very clear that the authors have undergone a very detailed thought process, but this is not always reflected in the written text.

Please take some time to review the text, make it consistent, and guide the reader along your line of arguments. What is your aim and who is your target audience?

I am certain these revision can help to strengthen your findings and conclusion.

Response: Thank you for your encouraging and supportive comments. We have reviewed the whole text and have made revisions to improve the readability.
